# Bridging Genomic Research Disparities in Osteoporosis GWAS: Insights for Diverse Populations

**DOI:** 10.1007/s11914-025-00917-2

**Published:** 2025-05-24

**Authors:** Qing Wu, Jingyuan Dai, Jianing Liu, Lang Wu

**Affiliations:** 1 Department of Biomedical Informatics, College of Medicine, The Ohio State University, 250 Lincoln Tower, 1800 Cannon Drive, Columbus, OH 43210 USA; 2 Pacific Center for Genome Research, University of Hawai’i at Mānoa, Honolulu, HI USA; 3 Population Sciences in the Pacific Program, University of Hawai’i Cancer Center, University of Hawai’i at Mānoa, Honolulu, HI USA

**Keywords:** Osteoporosis, Genome-wide association studies, Polygenic risk scores, Bone mineral density, Genetic disparities, Multi-omics

## Abstract

**Purpose of Review:**

Genome-wide association studies (GWAS) have significantly advanced osteoporosis research by identifying genetic loci associated with bone mineral density (BMD) and fracture risk. However, disparities persist due to the underrepresentation of non-European populations, limiting the applicability of polygenic risk scores (PRS). This review examines recent advancements in osteoporosis genetics, highlights existing disparities, and explores strategies for more inclusive research.

**Recent Findings:**

European-focused GWAS have identified key loci for osteoporosis, including *WNT* signaling (*SOST*, *LRP5*) and *RUNX2* transcriptional regulation. However, fewer than 40% of these variants can be replicated in Asian and African populations. Emerging studies in non-European groups reveal population-specific loci, sex-specific associations, and gene-environment interactions. Advances in machine learning (ML)-assisted GWAS and multi-omics integration are improving genetic discovery.

**Summary:**

Expanding GWAS in diverse populations, integrating multi-omics data, refining ML-based risk models, and standardizing biobank data are essential for equitable osteoporosis research. Future efforts must prioritize clinical translation to enhance personalized osteoporosis prevention and treatment.

**Supplementary Information:**

The online version contains supplementary material available at 10.1007/s11914-025-00917-2.

## Introduction

Osteoporosis is a major global health concern marked by low bone mineral density (BMD) and increased fracture risk. Each year, around 9 million fractures occur worldwide, with the burden expected to rise due to aging populations, especially in Asia and Latin America [1, 2]. Hip fractures, associated with a 24% one-year mortality rate and long-term disability, are projected to increase from 1.6 million in 2000 to 6.3 million by 2050, particularly in rapidly aging regions [1, 3]. In Europe, where osteoporosis affects 27.5 million individuals, healthcare costs are expected to exceed €37.5 billion by 2030 [1, 2]. Meanwhile, non-European populations are experiencing faster increases due to demographic transitions. For example, China is projected to see over 1 million hip fractures annually by 2050, while Latin America may face $13 billion in related healthcare costs [1, 4]. These trends underscore the need for population-specific prevention and treatment strategies.

Genome-wide association studies (GWAS) have transformed osteoporosis research, identifying over 500 genetic loci associated with BMD and fracture risk [5]. With up to 80% of BMD variability attributed to genetics, GWAS has elucidated key pathways like *WNT* signaling and transcriptional regulation by *RUNX2*, both crucial for bone remodeling and osteoblast differentiation [6–10]. Notable loci, including *SOST* and *LRP5*, have deepened our understanding of osteoporosis genetics and informed therapeutic advancements, such as sclerostin inhibitors and *WNT* modulators [11–17]. Several biomarkers demonstrate relevance across populations: *LRP5* mutations are associated with low BMD and higher fracture risk in both Asian and European groups [18–23] *COL1A1* and *COL1A2,* encoding type I collagen, are linked to osteogenesis imperfecta and general bone fragility, with the Sp1 polymorphism in *COL1A1* showing significant associations in both Asian and European cohorts [24–28]. Variants in the *VDR* gene, involved in calcium absorption via vitamin D signaling, are also associated with BMD variation across populations [29, 30]. These findings underscore the value of genetic insights for improving osteoporosis risk assessment and advancing personalized interventions globally.

Polygenic risk scores (PRS), which aggregate the effects of multiple genetic variants weighted by their GWAS-derived effect sizes, have shown promise in identifying individuals at high risk for osteoporotic fractures, particularly in European populations where most GWAS have been conducted [17, 31–34]. PRSs have successfully stratified fracture risk by integrating the cumulative impact of thousands of single-nucleotide polymorphisms (SNPs) associated with BMD [17, 35–37]. Despite recent advances, osteoporosis genetics research still faces notable disparities due to the underrepresentation of non-European populations in biobanks and GWAS, limiting the identification of population-specific variants and the clinical applicability of PRS in these groups [5, 38–40]. Less than 40% of BMD-associated SNPs discovered in Europeans are replicated in Asian populations, with similar challenges in African populations [38, 39]. Loci such as *SOST* and *WNT16* show population-specific effects, underscoring the need for more inclusive research efforts [41•, 42•, 43–45]. Additionally, gene-environment interactions involving diet and physical activity vary across cultures, complicating the generalizability of findings from European-centric studies [46, 47].

This review evaluates key GWAS discoveries in osteoporosis from 2021 to 2024, emphasizing recent findings, methodological innovations, and ongoing challenges such as limited genetic diversity and underexplored gene-environment interactions. By highlighting the need for inclusive biobanks, advanced analytical methods, and equitable research approaches, it supports progress toward personalized osteoporosis prevention and care.

## Recent Advances in GWAS for Osteoporosis

### Key Findings in European Populations

Advancements in GWAS have significantly improved the understanding of osteoporosis genetics in European populations by uncovering numerous loci and pathways that influence BMD and fracture risk. These studies have been instrumental in identifying genetic contributors and biological mechanisms essential for bone health.

#### Discoveries of Genes and Pathways

Large GWASs in European populations have identified many loci associated with BMD and fracture susceptibility. For example, multi-ethnic studies including European cohorts have identified loci such as *IGF2*, *ZNF423*, and *SIPA1* as contributors to BMD at different skeletal sites. Similarly, loci within the *WNT* signaling pathway, such as *SOST* and *LRP5*, have been shown to play critical roles in bone remodeling and osteoblast differentiation. Dysregulation of these pathways has been shown to impair bone formation, increase bone fragility, and play a role in developing osteoporosis in aging populations [48–50]. Notable findings include those involving *PDE11A* and *DKK2* [43, 51]*.* Further, regulatory pathways are important not only in bone development but also in lifelong bone maintenance and the progression of osteoporosis in later life (see Table 1).

**Table 1:** Summary of key genetic loci and pathways associated with osteoporosis risk across diverse populations (2021–2024)

Reference	Ancestry	Trait	Sample Size	Methodology	Key Findings	Highlights
**Yao et al. (2021)**[105]	European	BMD (FN, LS, heel) & serum urate interaction	*N* = 4,575 (FN), *N* = 4,561 (LS), *N* = 237,799 (heel)	GWAS	261 SNPs showed significant interaction with serum urate for BMD. Lead SNPs include **rs8192585, rs116080577.**	First gene environment GWAS for serum urate and BMD.
**Greenbaum et al. (2021)**[42•]	Multi-ethnic	BMD (FN, LS)	*N* = 44,426	GWAS meta-analysis	Identified **31 loci** for FN BMD and **30 loci** for LS BMD. Novel loci include **rs7111145, rs34290737.**	Multiethnic GWAS meta -analysis, including African American data.
**Younes et al. (2021)**[64•]	Qatari (Middle Eastern)	BMD (LS, pelvis, trunk)	*N* = 3,000	GWAS	15 variants significantly associated with BMD. **rs202070768** in MALAT1 was most significant (*p* = 1.30 × 10⁻⁹).	Qatari population study; validation of 5 UK Biobank loci.
**Barton et al. (2021)**[59]	European	BMD T-score	*N* = 445,855	GWAS	10 rare coding SNPs strongly correlated with BMD T-score (**rs142343894, rs144787122**).	WES-based GWAS; focuses on rare variants.
**Palmer et al. (2021)**[66]	African American	Volumetric BMD	*N* = 697 (T2DM)	GWAS	No genome wide significant SNPs after correction. **rs72354346, rs2024219** showed suggestive associations.	An important study in African American population with T2DM.
**Feng et al. (2021)**[68]	East Asian	BMD T-score	*N* = 150 (Discovery), N = 191 (Replication)	GWAS	Cluster of SNPs upstream of ***RREB1*** significantly associated with BMD (e.g., **rs473437**, *p* = 1.4 × 10⁻⁷)	Novel loci with larger effects on East Asians compared to Europeans.
**Sakaue et al. (2021)**[63•]	Multi-ethnic	Osteoporosis	*N* = 667,227 (488,501 European; 178,726 East Asian)	GWAS, GWAS meta-analysis	Significant differences in SNPs **rs12408050, rs34414754** between ancestries. European specific SNP: **rs144680237.**	Highlights genetic variability between European and East Asian cohorts.
**Tsai et al. (2021)**[85]	East Asian	Osteoporosis, Osteopenia	*N* = 651 (107 osteoporosis, 290 osteopenia, 254 controls)	Case control genetic association analysis	Identified 3 SNPs in the *RUNX2* binding site as candidate genes with **rs6086746** in the *PLCB4* promoter as a notable one. **rs6086746** AA allele increases osteoporosis risk in Chinese populations (OR = 6.89; 95% CI: 2.23–21.31, *p* = 0.001). A allele at rs6086746 reduces *RUNX2* binding affinity and suppresses *PLCB4* expression (*p* < 0.05).	Provided the genetic etiology of osteopenia and osteoporosis in Chinese postmenopausal women.
**Bae and Park (2022)**[69•]	East Asian	Osteoporosis	*N* = 8,842	GWAS	Identified SNPs in ***ADGRV1*** and ***PTPRD*** genes. Low calcium intake increased osteoporosis risk (OR = 2.07).	Demonstrated gene environment interaction with calcium intake.
**Choe et al. (2022)**[71]	East Asian	BMD	*N* = 5,111	PheWAS	Three SNPs identified: **rs7511649 C, chr1:150,441,950 T, rs1871859 T** (p < 1 × 10⁻⁶).	East Asian PheWAS for BMD traits.
**Lee et al. (2022)**[77]	East Asian	Bone stiffness index	*N* = 75,665	PheWAS	59 variants were found to be genome wide significantly associated with bone stiffness index (p < 5 × 10⁻^8^).	East Asian PheWAS for BMD traits.
**Auwerx et al. (2022)**[54]	European	BMD (heel)	*N* = 191,028	CNV GWAS	CNV at **16p11.2 BP4 BP5** associated with lower BMD. Overlap with SNP GWAS signals	CNV GWAS approach for osteoporosis.
**Fitzgerald and Birney (2022)**[55•]	European	Heel BMD, osteoporosis	*N* = 86,762	CNV GWAS	Fine mapped 4 CNVs for heel BMD (**cnvr125900, cnvr125930**). No significant signals for osteoporosis.	Focused on CNV contributions to heel BMD.
**Al Barghouthi et al. (2022)**[82]	European	eBMD (heel)	*N* = NA	TWAS, eQTL colocalization	Identified 512 genes, including ***RUNX2*, *IGF1*, *PPP6R3.***	Integrated transcriptomics and genetics for BMD.
**Zeng et al. (2023)**[41•]	East Asian	BMD (LS, total hip, FN)	*N* = 5,428	GWAS	12 loci associated with BMD; **rs79262027** (male specific) and **rs1239055408** (female specific).	First sex-specific GWAS in East Asians for BMD.
**Chen et al. (2023)**[62]	East Asian	BMD T-score, Z score	*N* = 102,900	GWAS, GWAS meta-analysis	Identified **968 novel loci** significant for BMD in East Asians. Lead SNP: **rs9379084** (*RREB1*).	Largest East Asian GWAS recently; loci not significant in Europeans.
**Li et al. (2023)**[16]	East Asian (T2DM)	LRP5 polymorphisms & bone metabolism	*N* = 226	Candidate gene study (LRP5)	**rs556442** associated with lower FN BMD (*p* = 0.013). AG/GG genotype linked to lower BMD and ALP levels.	Studied osteoporosis risk in T2DM postmenopausal women.
**He et al. (2023)**[70•]	Multi-ethnic	BMD (heel)	*N* = 147,123 (European), *N* = 5,862 (Asian)	GWAS	***CPED1-WNT16-FAM3C*** locus strongly associated with BMD trajectories in Europeans but not Asians.	Highlights longitudinal effects and ancestry differences.
**Qu et al. (2023)**[45]	European	SHBG and eBMD	*N* = 426,824	GWAS, Mendelian randomization	**219** shared loci between SHBG and eBMD. Increased SHBG levels were associated with decreased BMD (*p* = 3.04 × 10⁻^13^).	Novel overlaps between SHBG and bone health pathways.
**Jia et al. (2023)**[53]	European	BMD (FN, LS, forearm)	*N* = 52,236	GWAS	Identified **36** target regulatory genes BMD such as ***FAM3C*, *CCDC170*, *SOX6***, and ***PLEKHM1.***	An integrative GWAS analysis with regulatory SNPs for regulatory genetic variants detection.
**Xu et al. (2023)**[83]*	European	TB BMD	*N* = 10,414	TWAS	Identified 174 genes associated with BMD (*p* < 0.05) including ***IKZF1*** (*p* = 1.46 × 10^−9^) and ***CHKB*** (*p* = 8.31 × 10^−7^) in children. BMD associated genes revealed 200 gene ontology terms including protein catabolic process (log *p* = − 5.09) and steroid hormone mediated signaling pathway (log *p* = − 3.13).	TWAS and gene ontology analysis of TB BMD in children.
**Zhou et al. (2023)**[60]	Multi-ethnic	eBMD	*N* = 278,807 (European), *N* = 13,125 (African, East Asian, South Asian)	GWAS, WES	Rare coding alleles in 19 genes was associated with eBMD (*p* < 3.6 × 10^–7^) in Europeans. Consistent gene association was found in African, East Asian or South Asian ancestry including ***WNT5B*** and ***KREMEN1.***	Highlights the convergence of common and rare variants for bone therapeutic development using the CRISPR technique.
**Miao et al. (2024)**[61•]	European	BMD (FN, LS, TB)	*N* = 50,659	GWAS	Identified **188 loci**, including **89 novel loci.** ML increased discovery by 39% over conventional GWAS.	Machine learning enhanced GWAS for BMD traits.
**Su et al. (2024)**[65•]	Multi-ethnic	BMD (FN, LS, hip)	*N* = 2,875 (Caucasian), *N* = 2,107 (African American)	GWAS, TWAS, Epigenome	Identified **125 SV** associations for FN BMD**, 99** for LS BMD, and **83** for hip BMD. Identified **74** significant race specific SVs for African Americans. Identified **90** female specific and **129** male specific SVs for Caucasian and African Americans. Novel bone related genes including ***LINC02370***, *ZNF* family genes, and *ZDHHC* family genes. ***IBSP*** and ***SPP1*** as potential causal genes for osteoporosis	Sex- and ethnicity- stratified GWAS for SVs related to BMD traits.
**Lin et al. (2024)**[72]	East Asian	BMD	*N* = 86,716 (East Asian)	GWAS	Identified **78** significant SNPs and **75** genes related to BMD Highlighted pathways of **Hedgehog**, ***WNT* mediated**, and ***TGF ß.***	East Asian GWAS for BMD traits with highlighted pathways.
**Liaw et al. (2024)**[67]	East Asian	Osteoporosis	*N* = 29,084 (Discovery), *N* = 18,918 (Replication)	GWAS, GWAS meta-analysis, eQTL chromatin interaction mapping	**rs76140829** associated with osteoporosis (*p* = 1.15 × 10^–8^) Identified ***HABP2*, *RP11 481H12.1*,** ***RNU7 165P*, *RP11 139 K1.2*, *RP11 57H14.3*,** and ***RP11 214 N15.5*** genes in the positioning mapping of rs76140829.	East Asian GWAS for osteoporosis.
**Aparicio Bautista et al. (2024)**[75]	Hispanic	BMD (FN, LS, total hip)	*N* = 1,300 (Hispanic women)	GWAS	Significant association between **rs6086746 A** variant and FN BMD, LS BMD, and hip BMD. Interaction between **rs11623869 and rs2277458** was associated with total hip (*p* = 0.002) and FN BMD (*p* = 0.013).	Mexican women GWAS for BMD traits and vitamin D levels.
**Fouhy et al. (2024)**[76•]	Hispanic	BMD and Osteoporosis	*N* = 978 (Hispanic)	GWAS	**rs114829316, rs76603051, rs12214684,** and **rs77303493** were significantly associated with osteoporosis. **rs11855618** was associated with LS BMD; **rs73480593** was associated with total hip BMD. **7** SNPs showed a significant interaction effect with sugar sweetened beverages on osteoporosis.	Hispanic GWAS for osteoporosis and BMD related traits with gene diet interaction analysis.
**Wang et al. (2024)**[84]	Multi-ethnic	Osteoporosis	*N* = NA (European, African American), *N* = 1,539 (East Asian)	GWAS meta-analysis	**rs188303909** is a causal SNP for osteoporosis. **rs188303909** distally regulates *EN1* expression. *EN1* upregulates *CCDC170* and *COLEC10* expression through **rs4869739** and **rs4355801.**	Multi-ethnic GWAS analysis for osteoporosis.

Recent GWAS findings from 2021 to 2024 are shown with the ancestry of the populations, the corresponding traits, and the methodology used. Discovery and replication sample sizes, and ethnicities to conduct the GWAS, when available, are shown in the bracelets under the Sample Size column. Key Findings column summarizes major findings for each study including novel genetic variants, gene pathways, and functional analysis. Important studies are noted by• with reason under the Highlights column.

*GWAS* Genome wide association study, *PheWAS* Phenome wide association study, *TWAS* Transcriptome wide association study, *eQTL* Expression quantitative trait loci, *SHBG* Sex hormone binding globulin, *BMD* Bone mineral density, *FN BMD* Femur neck, *BMD LS BMD* Lumbar spine, *BMD, eBMD* Estimated, *BMD* *CNV* Copy number variation, *SV* Structural variant, *WES* Whole exome sequencing, *T2DM* Type 2 diabetes mellitus, *ALP* Alkaline phosphatase.

^*^log(*p*) is used in this study with no reporting of whether it represents the natural logarithm (ln) or the conventional − log₁₀(*p*)

Shared genetic pathways across diseases further illuminate osteoporosis risk. Liu et al. (2021) conducted a joint analysis of osteoporosis and rheumatoid arthritis (RA), identifying shared genetic loci that influence both bone and immune traits [52]. Their study revealed pleiotropic SNPs, such as rs13299616, have been linked to both BMD and autoimmune conditions such as RA, implicating genes such as *PTPN22* and *MAGI3* in bone metabolism and immune function [52, 53]. These findings underscore the shared biological mechanisms underlying osteoporosis and related conditions.

#### Methodological Innovations

Innovative methodologies have enhanced the precision and scope of GWAS in European populations, addressing the limitations of traditional approaches. Techniques such as CNV analysis, rare variant detection, and machine learning (ML), have revealed additional genetic contributors to osteoporosis risk:

1. **Copy Number Variation (CNV)-Based GWAS**: CNVs, large structural variations resulting in deletions or duplications of genomic segments, significantly contribute to BMD variability but are often overlooked in SNP-based analyses. CNV-based GWAS detects associations between these structural variations and traits or diseases, influencing gene expression and susceptibility. For example, CNVs at the 16p11.2 locus are associated with lower BMD, highlighting the importance of structural genomic variation in skeletal health (see Table 1) [54, 55•].
2. **Rare Variants**: Rare variant analyses have identified loci with stronger effect sizes than common variants. Whole-exome sequencing studies, for instance, have linked mutations in *COL1A2* and *EN1* to osteogenesis imperfecta, a monogenic disorder characterized by extreme bone fragility [56]. While osteogenesis imperfecta and osteoporosis are distinct conditions, rare deleterious variants in *COL1A2* are associated with reduced BMD and increased fracture risk in population-based studies [57–60], suggesting a potential overlap in genetic pathways contributing to bone strength and fragility.
3. **Machine Learning-Assisted GWAS**: ML frameworks enhance GWAS by integrating large-scale genomic and phenotypic data for improved genetic discovery and prediction. For example, POP-GWAS, which integrates imputed and observed phenotypes to identify novel BMD-associated loci, has improved the accuracy of genetic discovery by integrating observed and imputed phenotypic data. This approach has identified numerous novel loci, expanding the understanding of the genetic underpinnings of osteoporosis [61•].

### Insights from Non-European Populations

GWASs conducted in non-European populations have revealed important insights into the unique genetic architectures underlying BMD and fracture risk. These studies underscore the need for diverse representation to uncover population-specific loci and improve the applicability of genetic findings across populations.

#### Allele Frequency and Genetic Variation

Differences in allele frequencies and genetic variation contribute to disparities in GWAS findings between European and non-European populations. Variants with strong effects in one population may be insignificant or differ in effect size in others. For instance, Chen et al. (2023) identified 968 novel loci specific to East Asians, many of which were insignificant in Europeans, highlighting population-specific genetic heterogeneity [62]. Similarly, Sakaue et al. (2021) reported loci such as rs12408050 and rs34414754 with significant effects in Japanese but not European populations [63•]. These findings underscore the need to recalibrate tools like polygenic risk scores (PRS) for non-European populations, as applying European-derived models universally risks biased osteoporosis risk prediction.

#### Unique Genetic Loci and Effect Sizes

Loci identified in non-European populations often exhibit stronger or distinct effects than those observed in European populations. Younes et al. (2021) identified 15 BMD-associated variants in the *MALAT1* gene in the Qatari population [64•]. Su et al. (2024)[65•] identified novel genes like *LINC00494*, *ZNF* family genes, and *FMN2* in European-ancestry North Americans and African Americans. Palmer et al. (2021)[66] reported rs72354346 and rs2024219 associated with volumetric BMD in African Americans with T2DM. In East Asians, Liaw et al. (2024) identified rs76140829 in the *VTI1A* gene linked to osteoporosis in Taiwanese populations [67], and Feng et al. (2021) discovered SNPs upstream of *RREB1* associated with higher T-scores in Chinese postmenopausal women [68]. Similarly, Bae and Park (2022) identified SNPs such as rs7733007 in the *ADGRV* locus to be associated with BMD reduction in Korean participants [69•]. He et al. (2023) further illustrated differences in a longitudinal GWAS comparing European and Asian populations. For instance, the *CPED1-WNT16-FAM3C* locus, which showed a significant association in Europeans, was not replicated in Asians [70•]. Choe et al. (2022) conducted a phenome-wide association study (PheWAS), which is the inverse approach to GWAS that links a single genetic variant to multiple phenotypes and allows for the detection of pleiotropic effects and identified three SNPs associated with BMD in East Asians [71]. Yang et al. (2022) examined *WNT16* and *LRP5* polymorphisms in Chinese postmenopausal women, highlighting the protective effects of *LRP5* variants and the risk-increasing effects of *WNT16* under specific BMI conditions [15]. Li et al. (2023) further investigated *LRP5* polymorphisms in postmenopausal women with T2DM, linking specific genotypes to lower femoral neck BMD and emphasizing the role of metabolic traits in osteoporosis risk [16]. Lin et al. (2024) discovered SNPs and genes in the Taiwanese population linked to BMD and its correlation with metabolic traits like BMI and T2DM. They highlighted pathways including Hedgehog, *WNT*-mediated, and *TGF-β*. Bidirectional Mendelian randomization analyses indicated that higher BMI causally elevates BMD in this group [72]. These findings suggest that osteoporosis risk factors are not universally shared across populations, reinforcing the necessity for population-specific GWAS efforts. As PRS models continue to be integrated into clinical care, population-specific loci will be critical in refining risk prediction models for underrepresented populations.

#### Sex-Specific Associations

Sex-specific genetic associations with BMD have been previously reported, including in European children and adult populations [73, 74]. More recently, studies in non-European populations have also identified sex-specific associations. Zeng et al. (2023) identified loci such as rs79262027 in *VKORC1L1*, which was significantly associated with BMD exclusively in men, and rs1239055408 in *KCNJ2*, which was significant only in women [41•]. These findings suggest that genetic susceptibility to osteoporosis may be modulated differently between men and women across populations, suggesting that sex-specific PRS models may be needed to improve precision medicine approaches in osteoporosis risk assessment.

#### Gene-Environment Interactions

Gene-environmental interactions play a crucial role in shaping BMD in non-European populations. Bae and Park (2022) demonstrated that dietary calcium intake modulated the effects of variants in the *PTPRD* locus, with an increased risk of osteoporosis observed in Korean women with low calcium intake [69•]. Similarly, Aparicio-Bautista et al. (2024) highlighted gene-diet interactions involving vitamin D metabolism in Mexican Mestizo women, showing the protective effects of specific genetic variants against low BMD [75]. Fouhy et al. (2024) identified gene-diet interactions between rs72645876 in the *SNTG1* gene and the dietary approaches to stop hypertension diet, as well as interactions between seven SNPs and sugar-sweetened beverages in older Puerto Rican adults [76•]. These findings emphasize the necessity of integrating gene-environment interactions into osteoporosis risk assessment models, particularly in populations where dietary intake and lifestyle factors significantly modulate genetic effects.

#### Pleiotropic Effects and Disease Linkages

Pleiotropy further distinguishes genetic networks influencing BMD in non-European populations. Lee et al. (2022) identified loci associated with both glycemic traits and bone stiffness in Taiwanese populations, highlighting the importance of accounting for pleiotropic effects in genetic risk modeling [77]. Integrating PRS for osteoporosis with markers related to metabolic disorders, such as T2DM, may enhance the early identification of high-risk individuals. Future osteoporosis research should prioritize multi-omics approaches to comprehensively capture these pleiotropic relationships.

#### Genetic and Evolutionary Basis of BMD Differences

**Fig. 1:**
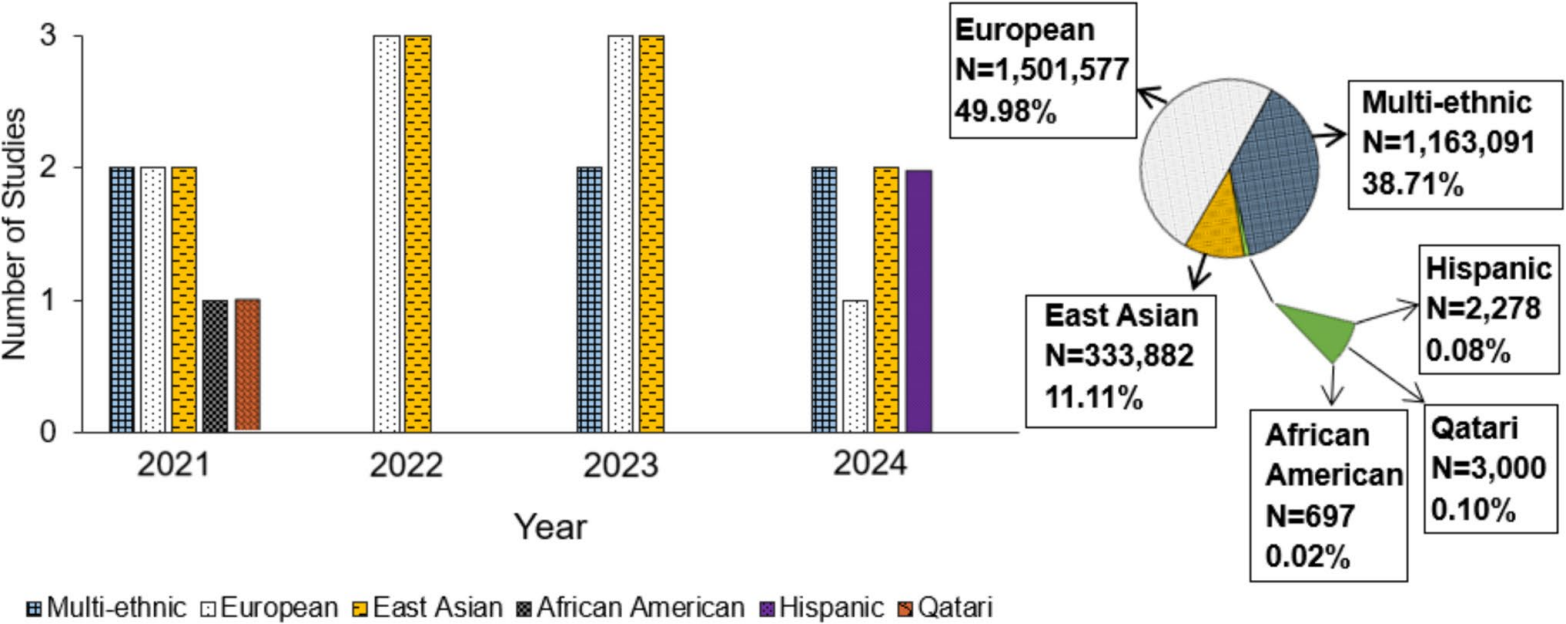
Osteoporosis GWAS Disparities: Annual Trends and Ancestry Representation (2021–2024). *The bar plot illustrates the distribution of osteoporosis GWAS studies by ancestry from 2021 to 2024, highlighting the disproportionate focus on European and East Asian populations. The pie chart shows the total sample size proportions across all years (N = 3,004,525), emphasizing the severe underrepresentation of Qatari, Hispanic, and African American populations (< 0.10%). These disparities underscore the need for more inclusive genetic research to improve osteoporosis risk prediction and treatment across diverse ancestries

Individuals of African ancestry typically exhibit higher BMD and lower fracture risk compared with those of European or East Asian ancestry [78, 79]. This difference is hypothesized to result from evolutionary pressures favoring greater bone mass and denser trabecular architecture, potentially as adaptive responses to historical environmental and mechanical loading conditions [80]. African populations also demonstrate greater genetic diversity at skeletal development-related loci, including higher frequencies of protective alleles associated with enhanced BMD, which may partially explain their reduced osteoporosis risk [81]. Despite the generally lower osteoporosis prevalence observed in African populations, the scarcity of GWAS and osteoporosis-focused genetic studies represents a significant gap in osteoporosis genomics research. The greater genetic heterogeneity of African populations complicates the identification of osteoporosis-related loci without sufficiently large sample sizes. Additionally, the lower clinical burden and fracture incidence in these populations may have led to less prioritization in research compared with European and East Asian populations (see Fig. 1).

#### Summary of GWAS Findings Across Populations

**Fig. 2:**
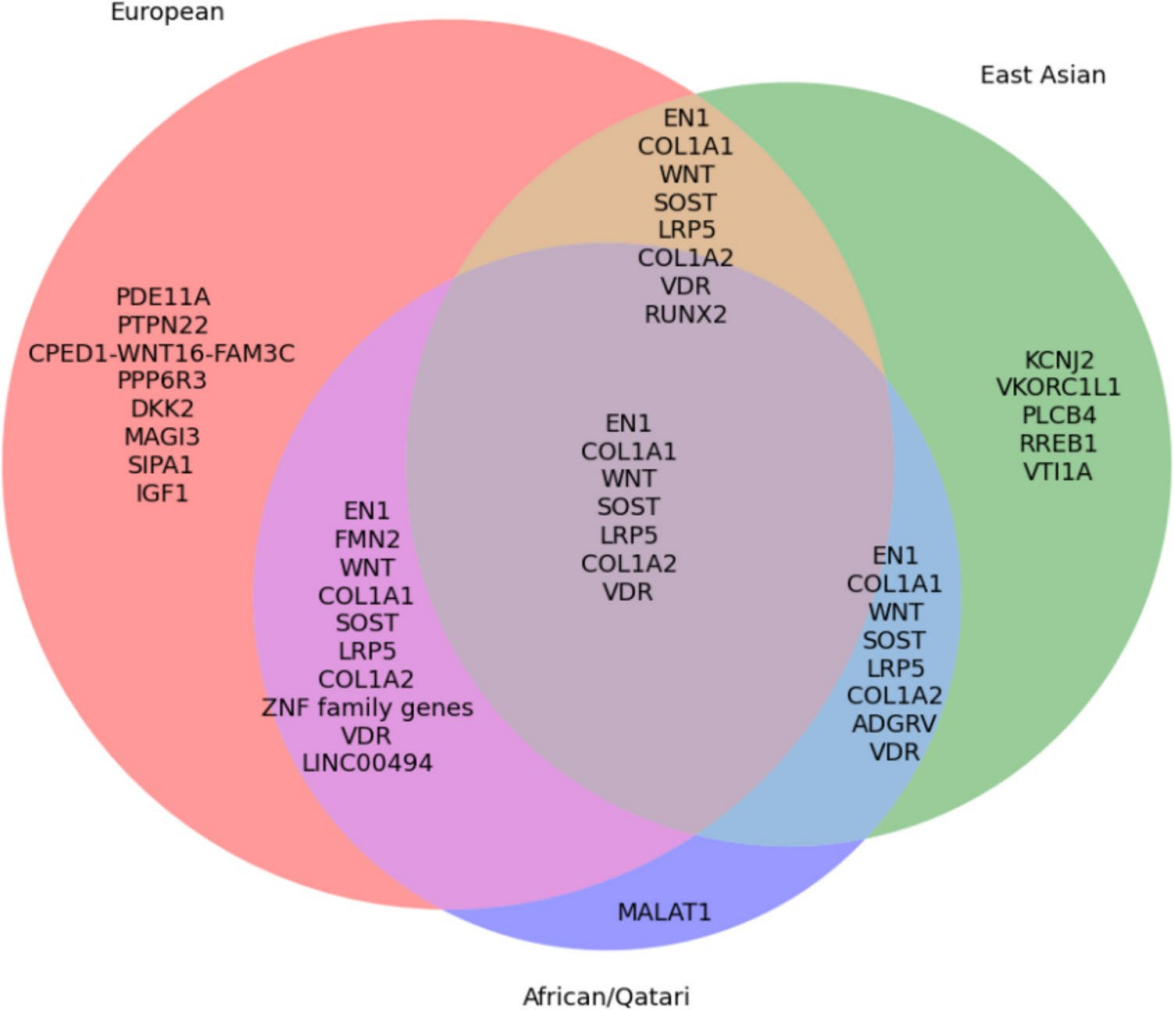
Overlap of Genetic Loci Associated with Osteoporosis Across Populations (2021–2024). *Several key loci, including *WNT*, *LRP5*, and *SOST*, are shared across European, East Asian, and African/Qatari populations. These loci are involved in the *WNT* signaling pathway. European-specific loci (*IGF1*, *SIPA1*, *DKK2*, *PDE11A*, *PTPN22*, *MAGI3*, *PPP6R3*) indicate pathways associated with collagen formation, osteoblast regulation, and hormonal signaling. East Asian-specific loci (*PLCB4*, *RREB1*, *VTI1A*, *VKORC1L1*, *KCNJ2*, *ADGRV*) suggest ancestry-driven genetic adaptations, possibly in response to environmental factors such as dietary calcium intake and bone metabolism variations. African/Qatari-specific loci (*MALAT1* and *ADGRV*) reveal potential novel regulatory elements affecting bone structure and BMD

GWASs in European populations have identified key loci such as *WNT16*, *LRP5*, and *SOST*, central to bone remodeling and osteoblast differentiation. The *WNT* signaling pathway remains a consistent regulator of BMD and fracture risk. Pleiotropic SNPs like rs13299616 link osteoporosis with autoimmune diseases, reflecting complex genetic networks. In contrast, studies in non-European populations reveal distinct architectures—e.g., rs473437 near *RREB1* in Chinese women and rs76140829 in *VTI1A* among Taiwanese. Unique loci in African American and Qatari populations (e.g., *MALAT1*, *ADGRV*) are absent or less impactful in Europeans, emphasizing the need for ancestry-specific risk models. (see Fig. 2) [76•]. Moreover, sex-specific variants like rs79262027 and rs1239055408 in East Asians support sex-stratified genetic assessments.

Population-specific GWAS findings offer insights into improving osteoporosis prediction and treatment. The absence of *CPED1-WNT16-FAM3C* significance in East Asians and the reduced *WNT16* effect size demonstrate limitations of applying European-based PRS universally. Additionally, gene-environment interactions—e.g., *PTPRD* variants with calcium intake in Koreans, and vitamin D metabolism genes in Mexican Mestizos—underscore the influence of lifestyle factors. Pleiotropic loci linking glycemia-related genes to BMD in Taiwanese cohorts further support integrating metabolic risk factors. Collectively, these findings reinforce the importance of diverse genetic studies and personalized models that incorporate ancestry, sex, and environmental factors in osteoporosis risk prediction.

### Functional Genomics

Functional genomics has greatly advanced our understanding of biological pathways underlying BMD regulation and osteoporosis risk by integrating transcriptomics, epigenetics, and proteomics data.

In European populations, functional studies have linked GWAS findings to key regulatory pathways. Al-Barghouthi et al. (2022) used a transcriptome-wide association study (TWAS), which integrates GWAS and gene expression data to identify genes whose genetically predicted expression levels are associated with disease risk, identifying 512 genes associated with BMD, including well-known regulators like *RUNX2* and *IGF1*, and novel candidates such as *PPP6R3*.[82] These findings underscore the critical roles of *RUNX2*, *IGF1*, and *PPP6R3* in osteoblast differentiation and bone microarchitecture, highlighting their potential as therapeutic targets. *RUNX2* is essential for skeletal development, and its dysregulation contributes to bone fragility. *IGF1* promotes osteoblast proliferation and supports peak bone mass. The identification of *PPP6R3* reveals novel regulatory mechanisms in skeletal maintenance; functional validation in mice showed that its deletion reduces lumbar spine BMD, suggesting it as a promising candidate for future drug development. Together, these genes provide insight into osteoporosis pathogenesis through their involvement in broader regulatory networks. Xu et al. (2023) identified 174 genes, such as *IKZF1* and *CHKB*, associated with total body BMD in children through TWAS and gene ontology analysis, emphasizing processes like protein catabolism and steroid hormone-mediated signaling pathways [83]. These findings underscore the importance of protein homeostasis and hormonal regulation in bone integrity, suggesting that targeting protein degradation pathways may offer therapeutic potential in osteoporosis prevention and treatment.

The pleiotropic effects of loci have also been explored. Qu et al. (2023) investigated shared loci between sex hormone‐binding globulin (SHBG) and eBMD, identifying 219 overlapping loci, including novel ones such as rs6542680 and rs815625 [45]. This study highlighted a causal relationship between SHBG levels and decreased BMD through Mendelian randomization analyses, which use genetic variants as instrumental variables to infer causal relationships between risk factors and the trait of interest. The identification of pleiotropic loci highlights the importance of multi-trait genetic analyses in osteoporosis research. For example, SHBG influences sex hormone regulation, affecting BMD by modulating bioavailable estrogen and testosterone, key hormones for skeletal strength, especially in postmenopausal women and older men. These findings suggest that hormone therapy or SHBG-targeted interventions may hold clinical promise for osteoporosis treatment. Similarly, Wang et al. (2024) identified a CpG-SNP, defined as a SNP’s allelic change introduced or removed a CpG site in the DNA sequence where cytosine is followed by guanine in a specific direction, at the 2q14.2 locus that regulates *EN1* expression via DNA methylation, impacting genes like *CCDC170* and *COLEC10*, which are implicated in bone formation [84]. Epigenetic modifications such as DNA methylation and histone changes reveal regulatory mechanisms influencing gene expression in bone formation, presenting promising targets for precision medicine in osteoporosis. Since these mechanisms respond to environmental and hormonal cues, factors like diet, exercise, and lifestyle may directly impact osteoporosis risk. Targeting epigenetic pathways through drugs or behavioral interventions offers a potential non-invasive approach to osteoporosis prevention.

Functional genomic studies in East Asian populations have revealed distinct loci and pathways with translational potential. Tsai et al. (2021) identified rs6086746, a polymorphism in the *PLCB4* promoter, as a key variant associated with osteoporosis risk. This variant could alter the binding affinity of *RUNX2*, a transcription factor essential for osteoblast differentiation, thereby affecting *PLCB4* expression [85]. These population-specific findings underscore the importance of inclusive research to identify genetic determinants missed in European-centric studies, supporting the need for global osteoporosis genomics efforts. The identification of ancestry-specific regulatory variants, such as those affecting *PLCB4*, emphasizes how genetic background influences bone metabolism. Given *PLCB4*’s role in calcium signaling, essential for osteoblast function, understanding its modulation across populations can inform more tailored osteoporosis therapies.

#### Shared and Population-Specific Pathways

Comparative studies between European and East Asian populations have uncovered both shared and unique genetic pathways. Conserved regulators like *RUNX2* and *IGF1* illustrate common mechanisms underlying bone health, while population-specific loci such as *PPP6R3* in European populations and rs6086746 in East Asian populations emphasize the importance of population-specific research. European studies have frequently highlighted pleiotropy and hormonal regulation, while East Asian studies have revealed loci with large effect sizes and gene-environment interactions, such as those influenced by BMI and dietary calcium intake [85].

## Methodological Challenges and Opportunities

Despite significant advancements in GWAS for osteoporosis, several methodological challenges persist, including limited transferability of findings, underrepresentation of non-European populations, and the integration of complex gene-environment interactions. Emerging solutions offer opportunities to address these issues and enhance the global applicability of osteoporosis genetics research.

### Challenges

#### Underrepresentation of Non-European Populations

A major challenge in osteoporosis GWAS is the lack of representation of non-European populations with the majority of recent studies focusing on European and East Asian populations, with severe underrepresentation of Hispanic, African American, and Qatari populations (see Fig. 1 and Supplement Table 1), which limits the applicability of findings across diverse populations (see Table 2 for key challenges and solutions). Many loci associated with BMD and fracture risk in Europeans, such as those near *EN1* and *COL1A2*, are rare or monoallelic in African, Asian, and Indigenous populations [41•, 42•, 43–45]. Fewer than 40% of BMD-associated SNPs identified in Europeans can be replicated in Asian populations, which may reflect both differences in genetic architecture and the limited statistical power of studies in non-European populations [42•]. These disparities constrain the clinical utility of PRS and other predictive tools, perpetuating inequities in osteoporosis risk prediction and management.

**Table 2:** Challenges and innovative solutions in GWAS

Challenges	Description	Proposed Strategies
Limited Sample Sizes in Diverse Populations	Underrepresentation of African, Asian, and Hispanic populations reduces statistical power and discovery of unique loci.	Recruit diverse cohorts through culturally tailored outreach and community engagement. Establish global consortia and funding incentives for the inclusion of underrepresented populations.
Lack of Data Standardization	Variability in phenotype definitions (e.g., BMD measurement techniques) and data collection protocols complicate meta-analyses.	Develop and adopt standardized protocols for BMD and fracture assessments across cohorts. Foster training programs for consistent data collection practices globally.
Data Sharing and Harmonization	Disparate data formats, access policies, and ethical concerns hinder cross population analyses.	Create centralized, secure repositories for data sharing. Develop harmonized pipelines for multi-ethnic meta-analyses. Promote equitable data sharing agreements.
Ethical Issues in Recruitment and Collaboration	Mistrust in research due to historical exploitation and concerns over data privacy in underserved populations.	Engage community stakeholders to build trust. Ensure culturally sensitive informed consent processes. Implement transparent data governance frameworks.
Transferability of Polygenic Risk Scores (PRSs)	PRSs developed in European populations often perform poorly in non-European groups due to differences in genetic architecture.	Develop ancestry specific PRS models. Train PRSs on multi-ethnic datasets to improve generalizability. Adjust weights for PRS's effect sizes based on ancestry specific allele frequencies and effect estimates to improve accuracy.
Integration of Gene Environment Interactions	Limited research on how lifestyle factors like diet and physical activity modulate genetic risk in diverse populations.	Conduct large scale, multi-ethnic longitudinal studies combining genetic and environmental data. Incorporate gene environment interaction terms in GWAS models.

#### Data Gaps and Limited Statistical Power

Biobanks and research cohorts often lack adequate representation of non-European populations, leading to smaller sample sizes and reduced statistical power to detect genetic associations [5]. African populations, which have the highest genetic diversity due to the"Out of Africa"migration, harbor unexplored loci critical for osteoporosis research [42•, 43]. Admixed populations, such as those in Latin America, present additional complexities due to mosaic genetic influences from Indigenous, African, and European populations. Expanding diverse biobank resources and leveraging trans-ethnic meta-analyses are essential to address these gaps (see Table 2).

### Opportunities

#### Inclusive Biobank Initiatives

Expanding biobank diversity is crucial for improving osteoporosis risk prediction in underrepresented populations. Key initiatives addressing this issue are summarized in Table 2. These efforts aim to develop population-specific reference panels and enhance the power of GWAS across diverse cohorts.

#### Standardized Protocols and Tools

Ensuring consistency across diverse datasets requires standardized data collection and bioinformatics tools (see Table 2 for key standardization efforts). Harmonized data collection practices, such as those promoted by the Global Biobank Meta-Analysis Initiative [86], improve reproducibility and facilitate data integration across global populations.

## Future Directions and Conclusion

Osteoporosis research has made significant strides in uncovering genetic factors underlying bone health through GWAS. However, to translate these findings into equitable clinical applications, several key areas warrant further exploration and strategic focus.

### Future Directions

1. **Increasing Diversity in GWAS Cohorts**

To address the persistent issue of underrepresentation, future research must prioritize expanding diverse cohorts and leveraging trans-ethnic meta-analyses. This effort includes developing new partnerships with global biobank initiatives and increasing recruitment efforts in underrepresented populations, such as the All of Us Research Program [87] and the Qatar Genome Project [88]. Such actions will enhance the discovery of population-specific loci and improve the generalizability of findings, ultimately creating more equitable PRS and genetic tools.

2. **Integrating Multi-Omics Approaches**

Integrating multi-omics data, including genomics, transcriptomics, proteomics, and metabolomics, provides a comprehensive approach for elucidating genetic influences on BMD. Multi-omics studies have identified pleiotropic effects and uncovered population-specific metabolic pathways, such as lipid metabolism differences between African and European populations [89]. These integrative strategies facilitate the identification of biomarkers relevant across diverse populations, thereby advancing personalized medicine for osteoporosis risk prediction and treatment [85]. State-of-the-art methods, including TWAS, chromatin accessibility assays, and single-cell RNA sequencing, enhance GWAS interpretation by linking genetic variants to regulatory mechanisms. For instance, TWAS has uncovered distinct regulatory genes influencing BMD in European and East Asian populations, highlighting its effectiveness in capturing population-specific genetic factors [90, 91]. Incorporating environmental and cultural data further enriches multi-omics approaches, providing deeper insights into gene-environment interactions affecting osteoporosis risk [63•]. Future studies should focus on combining multi-omics datasets with environmental and cultural factors to investigate these interactions comprehensively. Advances in single-cell sequencing and epigenomics offer promising opportunities to dissect regulatory mechanisms specific to underrepresented populations [92].

3. **Advancing Machine Learning Applications**

ML has emerged as a critical tool for overcoming the limitations of traditional GWAS methods by enhancing predictive accuracy, addressing population biases, and effectively utilizing multi-ethnic datasets characterized by different LD patterns and allele frequencies. Unlike conventional statistical approaches, ML algorithms handle high-dimensional genomic and phenotypic data, detect nonlinear relationships, and adaptively learn complex patterns, thereby improving osteoporosis risk prediction.

A significant challenge in GWAS, particularly in non-European populations, is the limited statistical power due to smaller sample sizes and distinct LD structures. Generative adversarial networks (GANs) offer a promising solution by generating synthetic genetic datasets that closely reflect real-world genomic variation, thus improving genetic association studies in underrepresented populations [93–95]. GAN-based approaches not only increase statistical power but also facilitate data augmentation strategies to enable variant discovery in populations with limited GWAS representation. Additionally, ML-driven fairness frameworks have emerged to identify and mitigate biases inherent in GWAS, ensuring equitable performance of PRS across populations. These models apply algorithmic bias correction techniques to improve PRSs' reliability in non-European populations [63•, 96]. Admixture mapping [97, 98], enhanced by ML approaches, provides specific advantages for genetically diverse populations such as Latin Americans and African Americans, where traditional GWAS may not capture ancestry-specific variants [63•, 96]. By leveraging differences in haplotype structures, ML-assisted admixture mapping identifies novel loci influencing BMD and fracture risk, thus contributing to more inclusive osteoporosis risk prediction models.

Integrating ML into GWAS is essential for advancing precision medicine, facilitating refined risk stratification, improving PRS portability, and enhancing the interpretability of complex genetic architectures. These advancements underscore the necessity for interdisciplinary collaboration, bridging genetics and computational science to develop generalizable and equitable genomic risk models for osteoporosis.

4. **Standardizing Data Collection and Analytical Pipelines**

Harmonizing data collection practices across diverse cohorts is vital to ensure reproducibility and comparability. Initiatives like the Global Biobank Meta-Analysis Initiative [86], the International HundredK + Cohorts Consortium (IHCC) [99], and the Musculoskeletal Knowledge Portal (MSK-KP) project[100] provide a framework for standardization and global collaborations. Future efforts should focus on establishing universally accepted protocols for phenotyping, genotyping, and environmental data collection, enabling robust cross-population analyses.

5. **Bridging Research and Clinical Implementation**

Translating GWAS findings into clinical practice requires bridging the gap between discovery and application [51]. This includes validating genetic findings in diverse cohorts, developing population-specific therapeutic targets, and creating user-friendly tools for clinicians to incorporate PRS into patient care [101]. Collaboration between researchers, clinicians, and policymakers will be crucial in ensuring that advances in genetic research benefit all populations equitably.

6. **Optimizing Osteoporosis Treatment Through Genetics**

Pharmacogenomics, which examines how genetic variation affects drug response, plays a pivotal role in optimizing osteoporosis therapies. Studies have shown that genetic variants influence responses to bisphosphonates, the most commonly used antiresorptive agents. Moreover, polymorphisms in estrogen metabolism-related genes, such as *PDSS1*, *CYP19A1*, *CYP1A1*, and *CYP1B1,* have been associated with varied efficacy of raloxifene, a selective estrogen receptor modulator frequently prescribed for postmenopausal osteoporosis [102].

Genetic ancestry significantly influences osteoporosis treatment, as population-specific differences in risk and drug metabolism necessitate ancestry-informed strategies. For example, *LRP5* variants affect bone formation and treatment response across populations [103]. Pharmacogenomic studies suggest that African and East Asian groups may benefit from adjusted dosing or alternative therapies due to unique genetic loci impacting BMD and drug metabolism [104].

Despite promising findings, pharmacogenomics remains underutilized in osteoporosis care. Future studies should prioritize large, ancestry-informed efforts to improve pharmacogenetic testing and enable more precise, safer treatments.

## Conclusion

GWAS has transformed osteoporosis research by uncovering key genetic factors underlying bone health. Despite this progress, critical challenges persist, including the underrepresentation of non-European populations and the limited integration of gene-environment interactions [69•, 76•, 105]. Addressing these gaps requires prioritizing diversity, expanding the use of multi-omics and machine learning, and standardizing global research practices [82, 106].

Future efforts should emphasize translating genetic findings into clinical tools that enhance osteoporosis prevention and treatment. Tackling current limitations will promote more equitable, data-driven approaches to improve bone health outcomes across diverse populations.

## Key References

- Zeng H, Ge J, Xu W, Ma H, Chen L, Xia M, et al. Twelve Loci Associated With Bone Density in Middle-aged and Elderly Chinese: The Shanghai Changfeng Study. J Clin Endocrinol Metab. 2023;108:295–305.
  - The first GWAS study focused on sex-specific effects in the East Asian population.
- Greenbaum J, Su K-J, Zhang X, Liu Y, Liu A, Zhao L-J, et al. A multiethnic whole genome sequencing study to identify novel loci for bone mineral density. Hum Mol Genet. 2021;31:1067–81.
  - A large-scale multi-ethnic GWAS including African Americans to identify novel loci associated with BMD.
- Fitzgerald T, Birney E. CNest: A novel copy number association discovery method uncovers 862 new associations from 200,629 whole-exome sequence datasets in the UK Biobank. Cell Genomics. 2022;2:100167.
  - A novel approach integrating CNV analysis with GWAS.
- He D, Liu H, Wei W, Zhao Y, Cai Q, Shi S, et al. A longitudinal genome-wide association study of bone mineral density mean and variability in the UK Biobank. Osteoporos Int. 2023;34:1907–16.
  - A longitudinal GWAS study focusing on European and Asian populations on heel BMD.
- Miao J, Wu Y, Sun Z, Miao X, Lu T, Zhao J, et al. Valid inference for machine learning-assisted genome-wide association studies. Nat Genet. 2024.
  - A novel approach to integrate machine learning techniques with GWAS.
- Sakaue S, Kanai M, Tanigawa Y, Karjalainen J, Kurki M, Koshiba S, et al. A cross-population atlas of genetic associations for 220 human phenotypes. Nat Genet. 2021;53:1415–24.
  - A large-scale GWAS on European and East Asian populations on osteoporosis and highlighted genetic variability between the two populations.
- Su K-J, Qiu C, Greenbaum J, Zhang X, Liu A, Liu Y, et al. Genomic structural variations link multiple genes to bone mineral density in a multi-ethnic cohort study: Louisiana osteoporosis study. J Bone Miner Res. 2024;39:1474–85.
  - A GWAS focusing on sex- and ethnicity-stratification analysis related to BMDs in European-ancestry of north American and African American populations.
- Bae J-H, Park D. Effect of dietary calcium on the gender-specific association between polymorphisms in the PTPRD locus and osteoporosis. Clinical Nutrition. 2022;41:680–6.
  - An interesting study focused on the gene-environment interaction of calcium intake and osteoporosis in the East Asian population.
- Younes N, Syed N, Yadav SK, Haris M, Abdallah AM, Abu-Madi M. A Whole-Genome Sequencing Association Study of Low Bone Mineral Density Identifies New Susceptibility Loci in the Phase I Qatar Biobank Cohort. Journal of Personalized Medicine. 2021;11:34.
  - A GWAS focusing on the Qatari population to identify variants associated with multiple BMDs including lumbar spine, pelvis, and trunk.
- Fouhy LE, Lai CQ, Parnell LD, Tucker KL, Ordovás JM, Noel SE. Genome-wide association study of osteoporosis identifies genetic risk and interactions with Dietary Approaches to Stop Hypertension diet and sugar-sweetened beverages in a Hispanic cohort of older adults. J Bone Miner Res. 2024; 39:697–706.
  - A GWAS for the Hispanic population on BMD and osteoporosis with gene-environment interaction analysis.

## Supplementary Information

Below is the link to the electronic supplementary material.

Supplementary file1 (DOCX 26 KB)

## Data Availability

No datasets were generated or analysed during the current study.
