# Supplementary material for: Bridging Genomic Research Disparities in Osteoporosis GWAS: Insights for Diverse Populations
Source: Curr Osteoporos Rep. 2025 May 24;23(1):24. doi: 10.1007/s11914-025-00917-2 (PMC12103327; doi:10.1007/s11914-025-00917-2)
Supplement: Supplementary file 1 — Supplementary file1 (DOCX 26 KB) [file 11914_2025_917_MOESM1_ESM.docx]

**Table 1. Major Cohorts Used in GWAS Associated with Osteoporosis Risk Across Diverse Populations (2021–2024)**

| Major Cohort | Multi-ethnic | European | East Asian | African American | Hispanic | Qatari |
| --- | --- | --- | --- | --- | --- | --- |
| BioBank Japan | * |  | * |  |  |  |
| UK Biobank | * | * |  |  |  |  |
| FinnGen | * |  |  |  |  |  |
| The Louisiana Osteoporosis Study | * |  |  |  |  |  |
| The Taiwan Biobank |  |  | * |  |  |  |
| The Insulin Resistance Atherosclerosis Family Study |  |  |  | * |  |  |
| The Boston Puerto Rican Health Study |  |  |  |  | * |  |
| The Qatar Biobank |  |  |  |  |  | * |

Major cohorts used in GWAS findings from 2021 to 2024 are shown, categorized by the ancestry of the populations included in each study. Asterisks (*) indicate which cohorts were utilized for each ancestry group. European and East Asian populations have well-established biobanks (UK Biobank, BioBank Japan, and the Taiwan Biobank), while African, Hispanic, and Middle Eastern populations remain underrepresented.
